# Supplementary material for: CATHe2: Enhanced CATH superfamily detection using ProstT5 and structural alphabets
Source: Biol Methods Protoc. 2025 Nov 4;10(1):bpaf080. doi: 10.1093/biomethods/bpaf080 (PMC12631783; doi:10.1093/biomethods/bpaf080)
Supplement: bpaf080_Supplementary_Data [file bpaf080_supplementary_data.docx]

**Supplementary Material:**

**Title:** CATHe2: Enhanced CATH Superfamily Detection Using ProstT5 and Structural

Alphabets

**Outline:**

This supplementary document provides more details on CATHe2 tested pLMs as well as preliminary experiments, hyperparameter fine tuning and training set modifications.


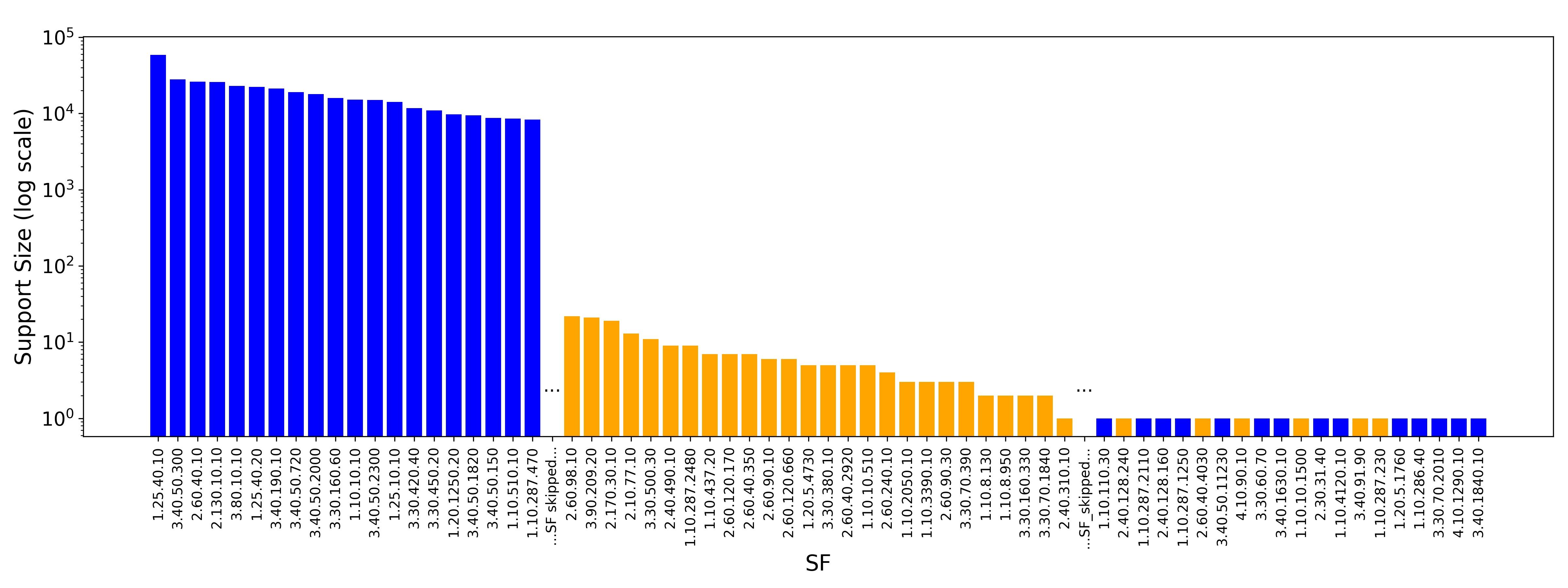


S1.Fig. 1: This bar plot shows the support sizes of some superfamilies in the CATHe training set, on a logarithmic scale. Orange bars represent all lost SFs for the 3Di sequence dataset, meaning the SFs that were lost due to unavailability of 3D structures for 3Di computation


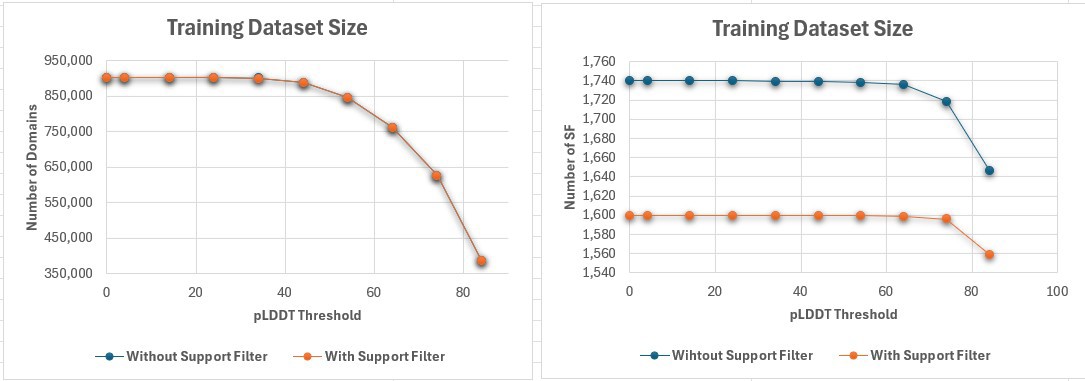


S1.Fig. 2. **Training set size evolution with the di**ff**erent filters.**

These plots show training set size for the different combinations of thresholds for the pLDDT filter and the support filter tested for CATHe2. The size is either counted with domain number or SF number.

**Tested pLMs, a more detailed presentation**

- ProtT5 (i.e, ProtT5-XL-U50) is the medium sized T5 encoder and the best-performing one from the ProtTrans paper [ref6]. This model is purely AA sequence based, meaning no structure information was used to train it. It was trained on BFD100 [ref79] and fine-tuned on Uniref50 [ref80]. Although ProtT5 embeddings were already used in CATHe, it is tested again for CATHe2 with a different code for generating embeddings, a better fine-tuned classifier architecture alongside 3Di embeddings in input. In this study ProtT5 proves again to be an excellent pLM for protein feature extraction by yielding very good results despite being rather old (2020).

- The ProstT5 pLM is a fine-tuned version of ProtT5 trained on both 3Di and AA span denoising task and bi-directional translation [ref8]. As a result, the encoder of ProstT5 can produce embeddings for both AA and 3Di sequences. The 3Di sequences used to train ProstT5 were computed from protein structures in AFDB [ref8, ref26]. ProstT5 is already being used in many works, often for 3Di prediction from primary sequences, bypassing full 3D prediction [ref23, ref67]. Moreover, in the ProstT5 paper, a CATH annotation benchmark is also carried out, using EAT and EAT+CL between embeddings from various inputs (AA, 3Di, ProstT5 predicted 3Di (p3Di), and the concatenation of AA and p3Di) comparing results with various pLM embeddings. The best results being consistently found to be with the ProstT5 pLM. In the CATHe2 code ProstT5 is called ProstT5 full as a half precision version called ProstT5 half is also tested for AA embedding computation (float16 weights instead of float32).

- TM-Vec is part of the tool duo TM-Vec+DeepBLAST whose purpose was to enable faster remote homology detection using structural alignment and deep learning [ref9]. TM-Vec produces structure-aware protein sequence embeddings, designed to help predict the TM-score [ref94], a measure of structural similarity between two protein sequences without the intermediate computation of their structures, even for remote homologs that fall below the 10% sequence identity. The model was trained on sequences from the CATH and SwissModel [ref95] structural databases. This model is actually based on ProtT5, with extra layers to help predict TM-scores [ref9, ref26]. For their CATH annotation benchmark, the - - TM-Vec team used EAT to find that TM-Vec separates CATH structural classes more clearly than the default ProtT5. Their results show that across each level of the CATH hierarchy, TM-Vec outperformed FoldSeek [ref7], MMseqs2 [ref38] and finally Prot-Tucker [ref22] which combines ProtT5 embeddings, EAT, and Contrastive Learning, for CATH annotation inference with HBI [ref22].

- ESM2 was introduced in 2023 alongside the ESMFold tool that predicts protein structure from AA sequences only [ref10]. ESM2 is a purely AA sequence based pLM with an attention mechanism to learn pairwise interactions between amino acid sequences. It was trained on the Uniref50 and Uniref90 databases to learn large amounts of information and representations from protein sequences using masked language modelling (MLM). The same model was trained on multiple scales, ranging from 8 million parameters to 15 billion parameters. It is the 15 billion parameter version that was tested in this study (also called ESM2 15B) as it was shown to perform better overall [ref10, ref26]. To fit this large model into the available memory space for CATHe dataset embedding, only a half precision version of ESM2 15B was tested (float16 weights instead of float32). This pLM was included in CATHe pLM tests as it was a recent pLM that had been shown to capture the primary sequence features related to 3D conformation. Plus it is very large and the proportionality between language model size and the richness of its learned representations has been validated in many fields [ref11], however, in this context, larger is not always better [ref6]. Even if ESM2 15B had shown incredible results, which is not the case (see Section Results in the main paper), using it for the final inference model of CATHe2 would not have been practical due to the enormous amount of memory required just to load it, even in half precision. To use such heavy pLMs in regular settings, memory efficient techniques have to be employed like the ones used in ESME [ref51].

- Ankh is a purely AA sequence based pLM using a T5-like architecture [ref6] with a 48-layer transformer trained with MLM on UniRef50 sequences [ref11, ref21, ref16]. According to the corresponding paper, Ankh is a pLM that focuses on protein-specific optimization rather than relying on large model size, i.e, involving empirical, knowledge-guided optimization to improve performance without a proportional increase in computational resources. There are actually two Ankh models that were tested in this project, Ankh large, which is the larger version of Ankh, and Ankh base which is smaller. Ankh was also benchmarked on a CATH annotation task and yielded rather good results compared to ESM2 and ProtT5, outperforming them for the mean accuracy across all CATH levels [ref11].

**Preliminary Experiments**

The first step of CATHe2 grid search fine tuning was to identify the “best” pLM to embed AA sequences with. This “best” pLM depends on the hyperparameters of course, that’s why a rather large range of hyperparameter combinations have been tested to try selecting the pLM with the most potential for further fine tuning. The next plots show some performance comparisons made during this step of preliminary experiments (during this first step the best combination of hyperparameters for ProstT5 full was obviously not found yet). The next step of preliminary experiments was to begin revealing clues about what the best values were for each hyperparameter, the next plots were also used to this effect.


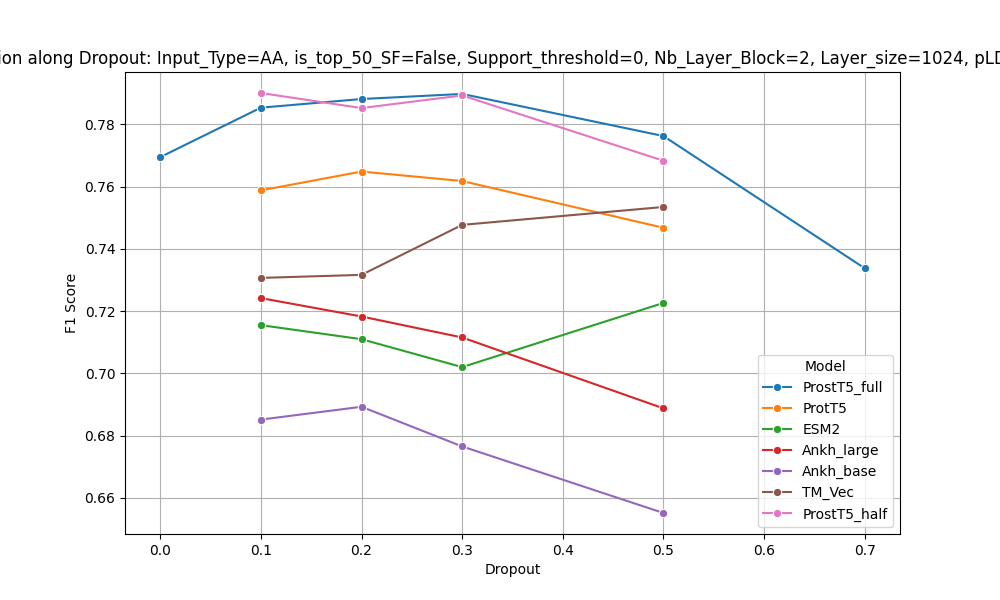


Hyperparameters: large dataset (is_top_50 = False)

pLDDT threshold: 0

support threshold: 0

input type: AA

Nb layer block: 2

Layer size: 1024


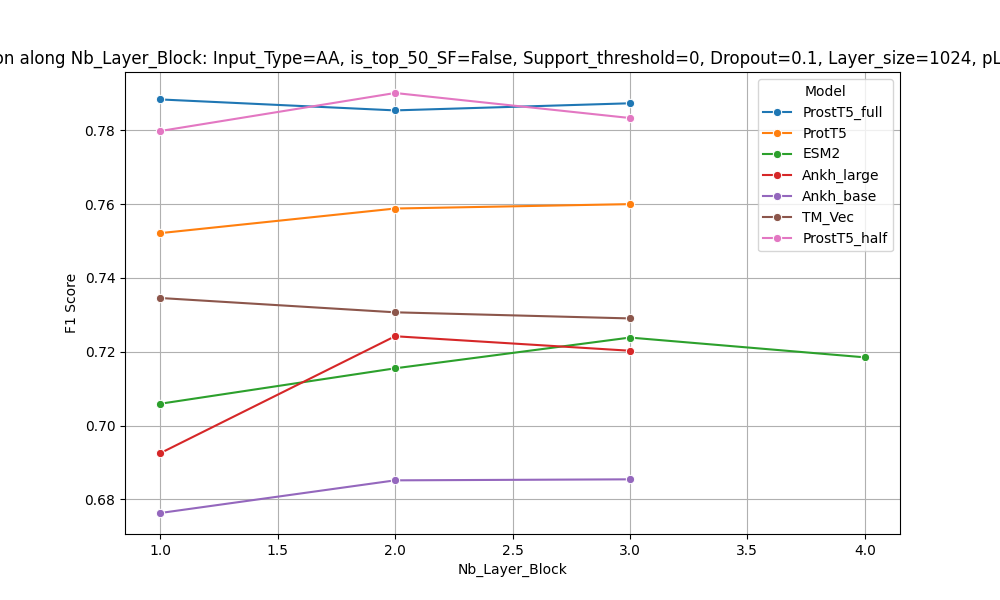


Hyperparameters: large dataset (is_top_50 = False)

pLDDT threshold: 0

support threshold: 0

input type: AA

Dropout: 0.1

Layer size: 1024


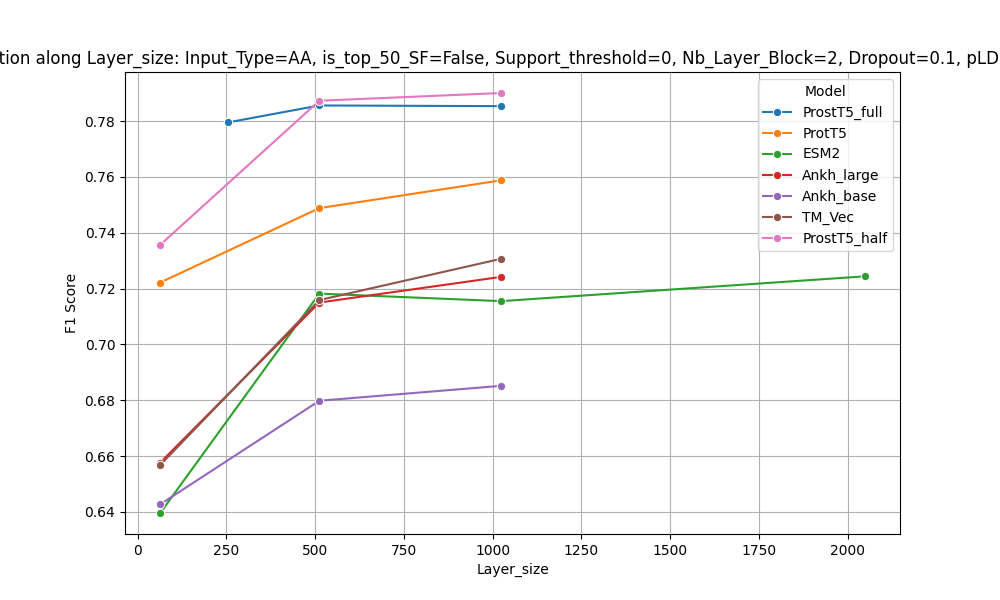


Hyperparameters: large dataset (is_top_50 = False)

pLDDT threshold: 0

support threshold: 0

input type: AA

Dropout: 0.1

Nb layer blocks: 2

**Hyperparameters fine tuning**

**Loss curve analysis**

As mentioned in the paper, the fine tuning of CATHe2 was conducted using a grid search guided by loss curve analysis. During CATHe2 model training, training loss and validation loss were saved to guide the next step of grid search. Here is an example of such a curves on a plot (this one is the loss curve of the final ProstT5 full model).


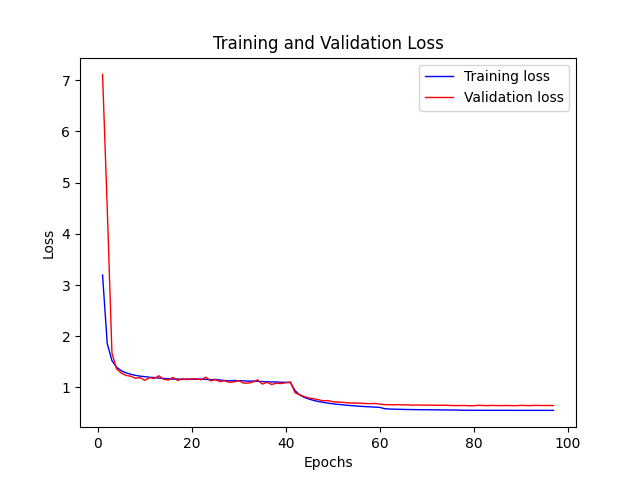


Much information can be deduced on such a plot. For instance an erratic loss curve can mean a too high learning rate. A validation loss getting close to training loss then separating from it can mean overfitting, which can be reduced by shortening epoch number or increasing dropout rate. A validation loss never approaching training loss can mean that the model does not have enough epochs to learn, or that the training set does not contain enough information to generalise, or that model structure is not complex enough to learn general classification rules. A too high training loss means that the model is not even learning classification rules for the training set, meaning this training set is not big enough or representative enough of classification rules, or that the model is not complex enough for example. All these clues and more can be derived from loss curves which gave some directions to explore more efficiently the hyperparameter space during grid search.

**Individual hyperparameter value analysis**

Model performance is linked to every single model hyperparameter in an unknown and probably non linear way. Thus trying to fine tune hyperparameters one by one is pointless, as for the same value of Dropout rate for example a large range of performance is possible depending on the other hyperparameters. All hyperparameters should be modified together to learn the right combination. However, it does not mean that nothing can be learned by analysing performance evolution when fixing all hyperparameters but one. It is expected for example that dropout rate should be increased when model complexity (number of layer blocks and dense layer size) is increased, to avoid overfitting. So learning the “right” dropout rate for a fixed model complexity can yield some indications about what is the “right” dropout rate to expect for other hyperparameter combinations. In addition, analysing performance along different hyperparameters, with all the other fixed can also yield clues about what performance to expect for other hyperparameter combinations, and what hyperparameter values are best. In the next plots for example, input type AA+3Di clearly performed better than any other input type. The following plots present some of the fine tuning process of ProstT5 full by showing performance evolution along a unique hyperparameter, while the others are fixed.


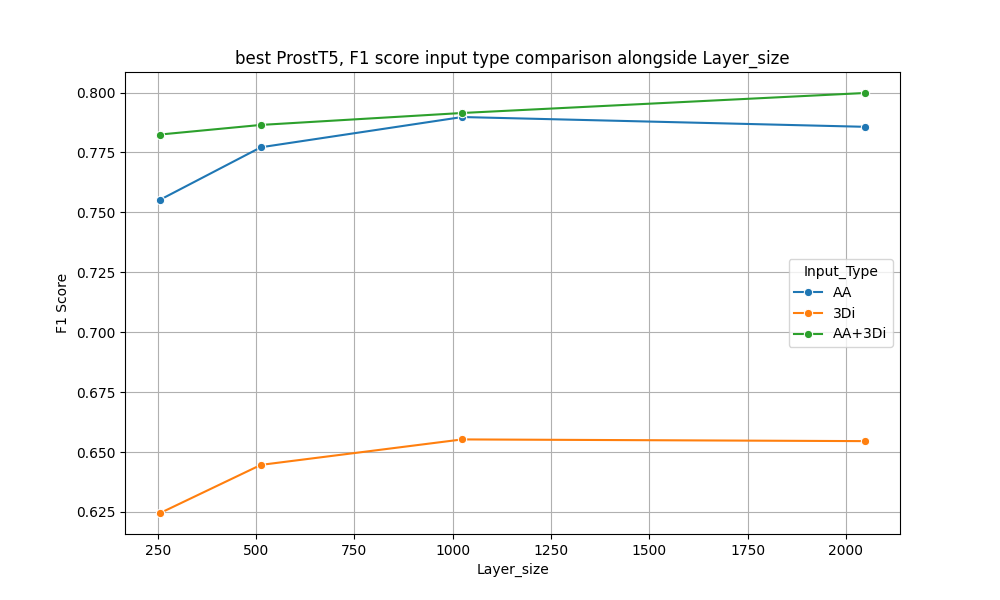


Hyperparameters: large dataset (is_top_50 = False)

pLM: ProstT5_full

pLDDT threshold: 0

support threshold: 0

Dropout: 0.3

Nb layer blocks: 2


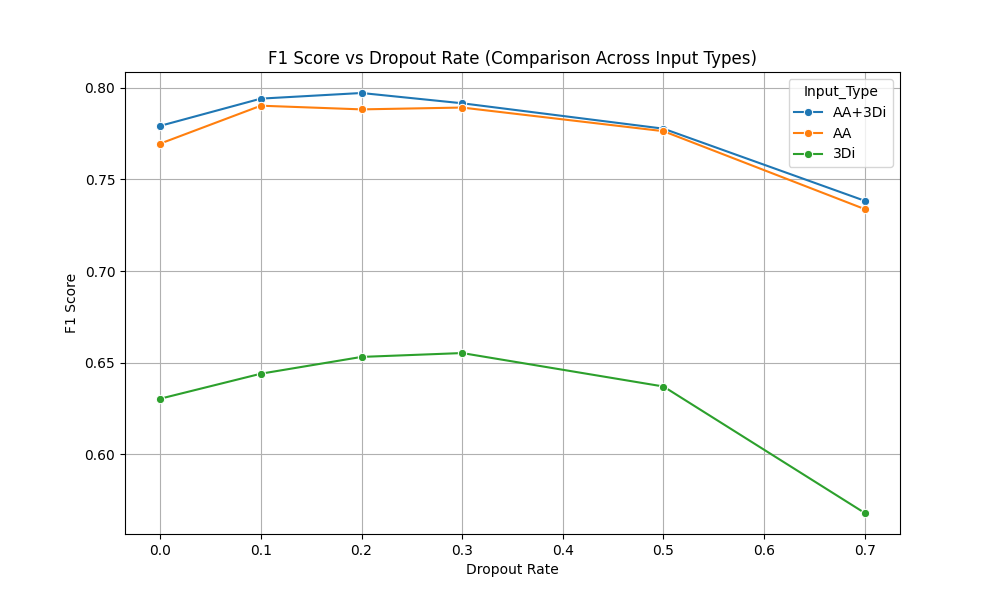


Hyperparameters: large dataset (is_top_50 = False)

pLM: ProstT5_full

pLDDT threshold: 0

support threshold: 0

Layer size: 1024

Nb layer blocks: 2


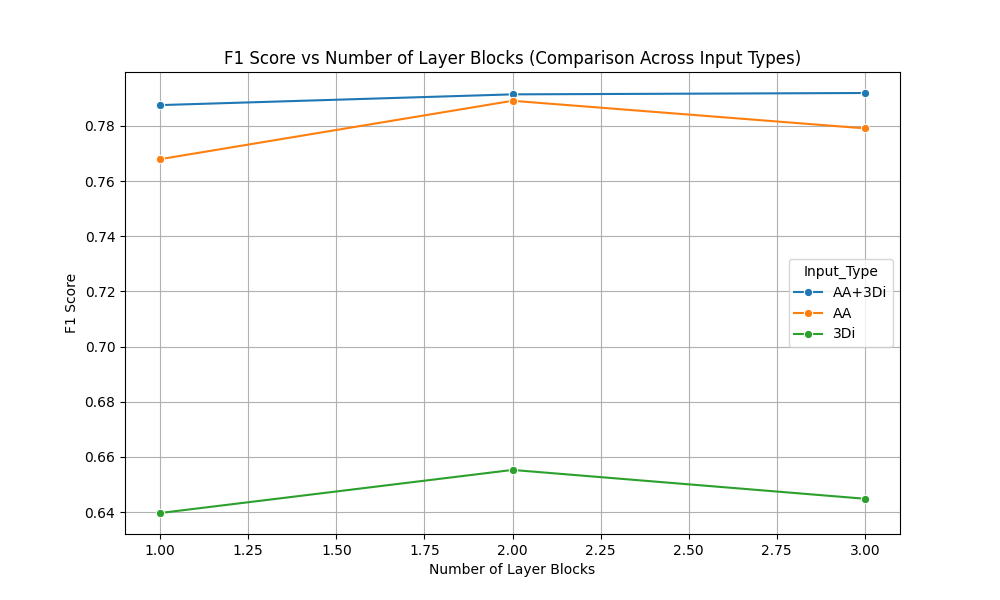
Hyperparameters: large dataset (is_top_50 = False)

pLM: ProstT5_full

pLDDT threshold: 0

support threshold: 0

Dropout: 0.3

Layer size: 1024

**Training set modifications relatively to CATHe2 filter thresholds**

| PLDDT  threshold | Lost SF count | Number of SF remaining | Training set size | Lost domains Count | Top 50 filtering | Support threshold |
| --- | --- | --- | --- | --- | --- | --- |
| 0 | 32 | 1,740 | 901,437 | 137,698 | FALSE | 0 |
| 4 | 32 | 1,740 | 901,437 | 137,698 | FALSE | 0 |
| 14 | 32 | 1,740 | 901,437 | 137,698 | FALSE | 0 |
| 24 | 32 | 1,740 | 901,429 | 137,706 | FALSE | 0 |
| 34 | 33 | 1,739 | 900,452 | 138,683 | FALSE | 0 |
| 44 | 33 | 1,739 | 887,929 | 151,206 | FALSE | 0 |
| 54 | 34 | 1,738 | 845,285 | 193 850 | FALSE | 0 |
| 64 | 36 | 1,736 | 762,164 | 276,971 | FALSE | 0 |
| 74 | 54 | 1,718 | 627,412 | 411,723 | FALSE | 0 |
| 84 | 125 | 1,647 | 388,478 | 650,657 | FALSE | 0 |
| 0 | 1,722 | 50 | 456,585 | 582,550 | TRUE | 0 |
| 4 | 1,722 | 50 | 456,585 | 582,550 | TRUE | 0 |
| 14 | 1,722 | 50 | 456,585 | 582,550 | TRUE | 0 |
| 24 | 1,722 | 50 | 456,585 | 582,550 | TRUE | 0 |
| 34 | 1,722 | 50 | 456,184 | 582,951 | TRUE | 0 |
| 44 | 1,722 | 50 | 449,897 | 589,238 | TRUE | 0 |
| 54 | 1,722 | 50 | 427,373 | 611,762 | TRUE | 0 |
| 64 | 1,722 | 50 | 384,638 | 654,497 | TRUE | 0 |
| 74 | 1,722 | 50 | 316,649 | 722,486 | TRUE | 0 |
| 84 | 1,722 | 50 | 192,677 | 846,458 | TRUE | 0 |
| 0 | 172 | 1,600 | 900,813 | 138,322 | FALSE | 10 |
| 4 | 172 | 1,600 | 900,813 | 138,322 | FALSE | 10 |
| 14 | 172 | 1,600 | 900,813 | 138,322 | FALSE | 10 |
| 24 | 172 | 1,600 | 900,805 | 138,330 | FALSE | 10 |
| 34 | 172 | 1,600 | 899,832 | 139,303 | FALSE | 10 |
| 44 | 172 | 1,600 | 887,321 | 151,814 | FALSE | 10 |
| 54 | 172 | 1,600 | 844,702 | 194,433 | FALSE | 10 |
| 64 | 173 | 1,599 | 761,630 | 277,505 | FALSE | 10 |
| 74 | 176 | 1,596 | 626,974 | 412,161 | FALSE | 10 |
| 84 | 213 | 1,559 | 388,204 | 650,931 | FALSE | 10 |

**References:**

ref6 : https://doi.org/10.1109/TPAMI.2021.3095381

ref7 : https://doi.org/10.1038/s41587-023-01773-0

ref8 : https://doi.org/10.1093/nargab/lqae150

ref9 : https://doi.org/10.1038/s41587-023-01917-2

ref10: https://doi.org/10.1126/science.ade2574

ref11: https://arxiv.org/abs/2301.06568

ref16: https://arxiv.org/abs/2501.10282

ref21: https://doi.org/10.3389/fbioe.2025.1506508

ref22: https://doi.org/10.1093/nargab/lqac043

ref23: https://www.biorxiv.org/content/early/2024/12/22/2024.12.22.629535

ref26: https://openreview.net/forum?id=IEZjjDX0iC

ref38: https://doi.org/10.1038/nbt.3988

ref51: https://doi.org/10.1101/2024.10.22.619563

ref67: https://hdl.handle.net/1992/74920

ref79: https://doi.org/10.1038/s41586-021-03819-2

ref80: https://doi.org/10.1093/bioinformatics/btu739

ref94: https://doi.org/10.1002/prot.20264

ref95: https://doi.org/10.1093/nar/gky427
